# Disparities in cardiac arrest mortality among patients with chronic kidney disease: A US‐based epidemiological analysis

**DOI:** 10.1002/joa3.13217

**Published:** 2025-01-10

**Authors:** Mahek Shahid, Hoang Nhat Pham, Ramzi Ibrahim, Enkhtsogt Sainbayar, Mahmoud Abdelnabi, Girish Pathangey, Amitoj Singh

**Affiliations:** ^1^ Department of Medicine University of Arizona Tucson Tucson Arizona USA; ^2^ Department of Cardiovascular Medicine Mayo Clinic Arizona Scottsdale Arizona USA; ^3^ Sarver Heart Center University of Arizona Tucson Tucson Arizona USA

**Keywords:** cardiac arrest, CKD, social vulnerability

## Abstract

**Background:**

Chronic kidney disease (CKD) increases cardiac arrest (CA) risk because of renal and cardiovascular interactions.

**Methods:**

Using Centers for Disease Control and Prevention (CDC) data from 1999 to 2020, we analyzed CKD‐related CA mortality and the impact of social vulnerability index (SVI).

**Results:**

We identified 336 494 CKD‐related CA deaths, with stable age‐adjusted mortality rates over time. Disparities were observed across gender, racial/ethnic, and geographic subpopulations, with higher mortality among males, Hispanic and non‐Hispanic Black populations, and those in urban and Western regions. Higher SVI correlated with increased mortality.

**Conclusions:**

CKD‐related CA mortality rates are stable, with disparities across demographics; higher SVI correlates with increased mortality, highlighting needed interventions.

## Graphical abstract

CKD‐related CA mortality rates have remained stable with temporary rise in the early 2010s. Disparities in CKD‐related CA mortality were observed among gender, racial/ethnic, socioeconomic status, and geographic subpopulations

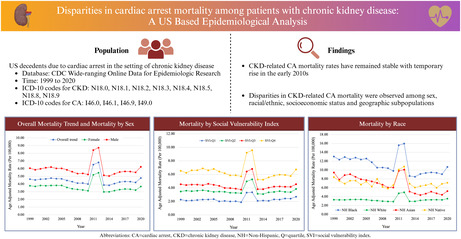

## 1 INTRODUCTION

Chronic kidney disease (CKD) increases the risk of cardiac arrest (CA) because of the interplay between renal dysfunction and cardiovascular complications. As CKD progresses, the incidence of CA rises, contributing to 60% of cardiac deaths among patients undergoing dialysis.^1^, ^2^ The pathophysiology underlying this elevated risk is multifactorial, involving electrolyte imbalances, sympathetic overactivity, inflammation, and iron deposition, all of which can predispose to arrhythmias and conduction abnormalities.^1^ Structural heart changes and electrophysiological alterations also contribute to the increased risk of malignant ventricular arrhythmias and CA in patients with CKD.^3^

Social determinants of health (SDoH) further complicate morbidity and mortality associated with kidney disease and CA. SDoH are the conditions in which people are born, grow, live, work, and age, which can significantly impact health outcomes. These determinants include nonmedical factors, such as education, economic stability, healthcare access and quality, social and community context, neighborhood and built environment, and systemic biases.^4^ The social vulnerability index (SVI), a SDoH tool developed by the Centers for Disease Control and Prevention (CDC), has been shown to impact cardiovascular disease prognosis.^4^ This tool is a quantitative measure that includes characteristics of population‐level socioeconomic status, household characteristics, racial/ethnic minority status, and housing type and transportation. Our study evaluated disparities and impact of the SVI on CA mortality related to CKD in the United States (US).

## 2 METHODS

We queried US mortality and demographic data between 1999 and 2020 from CDC Wide‐ranging Online Data for Epidemiologic Research (CDC WONDER) database utilizing ICD‐10 codes CKD (N18.X) and CA (I46.X, I49.0) within the multiple causes‐of‐death files. The CDC WONDER database is a comprehensive public health resource that compiles data from national surveys, vital statistics, and disease registries, capturing over 99% of all deaths in the United States to enable the analysis of population health trends.^5^ As defined by ICD‐10 code, CKD is classified as N18.X and refers to a disorder marked by the gradual and typically permanent loss of kidney function, leading to renal failure. CA is defined by two ICD‐10 codes, I46.X and I49.0, which refers to a disorder characterized by cessation of the pumping function of the heart.^6^ Age‐adjusted mortality rates (AAMR) per 100 000 and 95% confidence intervals were estimated using the Direct Standardization method, standardized to the 2000 US population.

The CDC's SVI measures the vulnerability of communities by analyzing 16 social factors grouped into four main domains. These domains capture key aspects, such as socioeconomic status, household composition, minority status and language, and housing type and transportation. Each area of residence is assigned a percentile ranking for each of the four domains and an overall combined ranking (Table 1). The scores range from 0 to 1, where 0 indicates the least socially vulnerable communities and 1 indicates the most socially vulnerable. The SVI is based on 5‐year estimates of data collected from the American Community Survey.^4^ The 2020 SVI data were categorized into four quartiles, from the lowest Q1 (least vulnerable) to the highest Q4 (most vulnerable) and linked to mortality data using county codes. Log‐linear regression was used to analyze temporal trends of AAMR by identifying the fewest significant joint points. Mortality between Q1 and Q4 SVI quartiles in the overall population was compared by logistic regression. Average annual percentage change (AAPC) was calculated by averaging of the annual percentage changes (APC), which were determined through Monte‐Carlo permutation tests. We estimated the excess AAMR attributable to COVID‐19 by comparing the projected AAMR for 2020, calculated based on 1999–2019 trends, with the actual AAMR in 2020. Two‐tailed *t*‐tests were utilized to assess significant changes in the APC, with *p* < .05 indicating significance. Institutional Review Board approval was not needed as it involved deidentified and publicly available data regarding nonliving humans.

**TABLE 1:** Social vulnerability index. The 16 social characteristics, under four themes, that are included in the social vulnerability ranking system.

Housing type and transportation	Racial and ethnic minority status (one of the below)	Household characteristics	Socioeconomic status
Crowding	Hispanic or Latino (of any race)	Aged 17 or younger	No health insurance
No vehicle	Native Hawaiian and Other Pacific Islander	Aged 65 or older	Housing cost burden
Mobile homes	American Indian and Alaska Native	Single‐parent households	Unemployed
Multi‐unit structures	Black and African American	English language proficiency	Below 150% poverty
Group quarters	Other races (not Hispanic or Latino)	Civilian with a disability	No high school diploma

## 3 RESULTS

A total of 336 494 CA deaths in the setting of CKD were identified. AAMR remained steady at 4.64 (95% CI, 4.56–4.72) in 1999 to 4.79 (95% CI, 4.72–4.86) in 2020, despite an isolated AAMR spike to 6.82 (95% CI, 6.74–6.91) between 2011 and 2012 (Graphical abstract). Cumulative AAMR during our 22‐year study period was 4.57 (95% CI, 4.56–4.59), with an AAPC of −0.26% (95% CI, −1.46–0.96, *p* = .66).

Males (AAMR 5.95 [95% CI, 5.92–5.98]) had higher mortality than females (AAMR 3.60 [95% CI, 3.58–3.62]). Mortality was higher among Hispanic populations (AAMR 7.54 [95% CI, 7.46–7.61]) compared with non‐Hispanic (NH) populations (AAMR 4.32 [95% CI, 4.30–4.34]). Mortality was highest among NH Black populations (AAMR 11.03 [95% CI, 10.95–11.11]), followed by NH American Indian/Alaska Native (AAMC 7.10 [95% CI, 6.83–7.37]), NH Asian/Pacific Islander (AAMR 6.32 [95% CI, 6.23–6.42]), and NH White (AAMR 3.38 [95% CI, 3.36–3.39]) populations. Notably, the CA‐related mortality in NH Black population with CKD (AAPC −1.56% per year, *p* = .01) declined significantly between 1999 and 2020, compared with their White counterparts (AAPC +0.18% per year, *p* = .75).

Mortality was higher among urban regions (AAMR 4.76 [95% CI, 4.74–4.77]) than rural regions (AAMR 3.70 [95% CI, 3.67–3.73]). Western US regions (AAMR 6.70 [95% CI, 6.66–6.74]) had the highest mortality rates in the United States, followed by Northeastern (AAMR 5.53 [95% CI, 5.49–5.57]), Southern (AAMR 3.96 [95% CI, 3.94–3.99]), and Midwestern (AAMR 2.61 [95% CI, 2.58–2.63]) regions. Mortality trends are presented in Table 2. Mortality in Q4 (AAMR 6.39 [95% CI, 6.36–6.42]) was higher compared with Q1 (AAMR 2.30 [95% CI, 2.27–2.33], *p* < .001), with higher SVI accounting for 4.09 excess deaths per 100 000 person‐years. During the first pandemic year, deaths exceeded projections by 12.43%, with notable increases in females (13.25%), Hispanic (16.93%), NH Asian (18.74%), and NH Black (16.80%) population with CKD, as well as those in urban areas (12.60%) and the Northeast region (14.11%) (Table 3).

**TABLE 2:** Joinpoint analysis. CA mortality trends in population with CKD in the United States between 1999 and 2020.

Joinpoint segment	Duration	APC (95% CI)	APC *p* value	AAPC (95% CI)	AAPC *p* value
Overall					
1	1999–2020	−0.26 [−1.46–0.96]	.66	−0.26 [−1.46–0.96]	.66
Gender					
Female					
1	1999–2020	−0.47 [−1.76–0.85]	.47	−0.47 [−1.76–0.85]	.47
Male					
1	1999–2020	−0.18 [−1.28–0.94]	.74	−0.18 [−1.28–0.94]	.74
Ethnicity					
Hispanic					
1	1999–2020	−2.23 [−3.61 to −0.83]	.003	−2.23 [−3.61 to −0.83]	.003
Non‐Hispanic					
1	1999–2020	−0.26 [−1.36–0.86]	.63	−0.26 [−1.36–0.86]	.63
Race					
NH Black					
1	1999–2020	−1.56 [−2.70 to −0.40]	.01	−1.56 [−2.70 to −0.40]	.01
NH White					
1	1999–2020	0.18 [−0.99–1.37]	.75	0.18 [−0.99–1.37]	.75
NH Asian					
1	1999–2009	−3.80 [−5.42 to −2.15]	<.001	−2.85 [−6.32–0.75]	.12
2	2009–2012	13.26 [−5.90–36.32]	.17		
3	2012–2015	−24.03 [−37.77 to −7.24]	.01		
4	2015–2020	4.74 [0.68–8.96]	.03		
NH native					
1	1999–2020	−0.52 [−1.75–0.72]	.39	−0.52 [−1.75–0.72]	.39
Census region					
Northeast					
1	1999–2020	−1.03 [−1.94 to −0.10]	.03	−1.03 [−1.94 to −0.10]	.03
Midwest					
1	1999–2020	1.49 [0.27–2.74]	.02	1.49 [0.27–2.74]	.02
South					
1	1999–2020	−0.33 [−1.34–0.70]	.51	−0.33 [−1.34–0.70]	.51
West					
1	1999–2020	−0.47 [−2.03–1.12]	.55	−0.47 [−2.03–1.12]	.55
Urbanization					
Urban					
1	1999–2020	−0.32 [−1.57–0.95]	.60	−0.32 [−1.57–0.95]	.60
Rural					
1	1999–2020	0.63 [−0.39–1.67]	.21	0.63 [−0.39–1.67]	.21
Social vulnerability index					
Quartile 1					
1	1999–2020	0.74 [−0.49–1.99]	.22	0.74 [−0.49–1.99]	.22
Quartile 2					
1	1999–2020	0.08 [−0.96–1.13]	.88	0.08 [−0.96–1.13]	.88
Quartile 3					
1	1999–2020	−0.13 [−1.36–1.13]	.84	−0.13 [−1.36–1.13]	.84
Quartile 4					
1	1999–2020	−0.34 [−1.54–0.87]	.56	−0.34 [−1.54–0.87]	.56

Abbreviations: AAPC, average annual percentage change; APC, annual percentage change; CA, cardiac arrest; CI, confidence interval; CKD, chronic kidney disease; NH, non‐Hispanic; SVI, social vulnerability index.

**TABLE 3:** Estimated excess death. Estimated excess death and percentage excess mortality rate in 2020 in comparison with 1999–2019 period.

	AAPC 1999–2019	Projected AAMR 2020	Actual AAMR 2020	% excess AAMR 2020	Estimated excess death 2020
All	−0.37	4.19	4.79	12.43%	2458
Female	−0.55	3.19	3.68	13.25%	1130
Male	−0.29	5.50	6.24	11.79%	1327
Hispanic	−2.46	5.76	6.94	16.93%	485
Non‐Hispanic	−0.34	4.03	4.55	11.51%	1940
NH Black	−1.74	8.90	10.7	16.80%	742
NH White	0.12	3.28	3.55	7.49%	835
NH Asian	−3.48	4.27	5.25	18.74%	207
NH Native	−0.58	6.85	7.14	4.06%	8
Urban	−0.41	4.28	4.9	12.60%	2136
Rural	0.50	3.82	4.28	10.77%	304
NE	−1.22	4.87	5.67	14.11%	619
Midwest	1.28	2.96	3.38	12.50%	368
South	−0.37	3.54	3.96	10.69%	660
West	−0.52	5.93	6.75	12.16%	764

## 4 DISCUSSION

Our analysis of CKD and CA identified disparities based on gender, race/ethnicity, and geographic populations, with SVI significantly contributing to excess mortality. The composite temporal trend in mortality remained stable over time, contrasting with the declining trend of CA‐related mortality observed in the general population.^7^ This may reflect insufficient adoption of risk factor management and low utility of standardized CKD management in vulnerable populations, despite advancements in guidelines over the last two decades.^8^ The temporary rise in CKD‐related CA mortality during the early 2010s could be attributable to healthcare access barriers, such as those following the 2008 financial crisis, before improvements under the 2010 Patient Protection and Affordable Care Act.^9^, ^10^ Prior epidemiological data has shown CKD is more prevalent in females, but experience less CVD than males at all stages of CKD.^11^ Our analysis revealed that males with CKD had 65% higher mortality from CA compared with females. This disparity may result from more rapid progression of CKD in males,^12^ who also have a greater burden of CKD risk factors like hypertension.^11^

Excess mortality related to social vulnerability aligns with previous studies, such as the REGARDS sub‐study, which showed that household poverty was independently associated with kidney disease across both Black and White populations.^13^ Additionally, Black and Hispanic individuals with CKD have lower life expectancy than their White counterparts, often due to neighborhood socioeconomic status and limited access to resources, such as hemodialysis facilities or health insurance.^14^ This disparity leads to delay in diagnosis and treatment which could exacerbate comorbidities and increase the risk of CVD.^15^ Additionally, genetic variations also play a significant role in the racial disparities in CA‐related mortality among Hispanic and Black individuals with CKD. For example, variants in the apolipoprotein L1 (APOL1) gene have been shown to confer risk for CKD development and progression. Black patients with two APOL1 risk alleles have a significantly higher risk of CKD progression and end‐stage renal disease (ESRD) compared to those with zero or one risk allele, with a 1.49‐fold increased risk of CKD and a 1.88‐fold increased risk of ESRD.^16^, ^17^ Other genetic factors associated CA, such as ion channelopathies and inherited cardiovascular diseases, also exhibit racial disparities, with African American/Black individuals being disproportionately affected.^18^ Interestingly, our analysis showed that the CA‐related mortality gap between Black and White patients with CKD during study period, mainly stemming from significant decline in mortality in Black populations. This overall decreasing trend was likely due to increased health access, which promotes better management of risk factors, earlier detection of CKD, and long‐term management of this chronic disease. Shifts in the insurance market have also allowed for more patients to qualify for dialysis treatments via expanded private or public insurance coverage.^19^

Urban communities experienced greater CA mortality compared with rural communities, likely due to urban infrastructure that fails to promote physical activity and increased environmental stressors. This may lead to poor cardiometabolic heath in disadvantaged neighborhoods within metropolitan regions.^20^, ^21^ On the contrary, rural communities have been shown to have reduced access to health care and delays in evaluation and treatment.^22^ Prior study showed that rural patients with out‐of‐hospital CA have longer emergency medical services response times, lower odds of achieving return of spontaneous circulation and survival to hospital discharge compared with urban patients.^23^ However, pockets within urban communities face this same desert of healthcare facilities compounded with income inequality, decreased social support due to residential structure, increase noise, and air pollution, as well as decreased sleep quality all contributing to increased cardiovascular risk and poor outcomes.^24^ Despite numerous studies showing that Southern US region bears a heavier CVD burden and subsequent cardiovascular mortality,^25^ our findings revealed that the Western US regions experienced the highest mortality rates. This emphasizes the necessity for further investigation to understand the connection of lifestyle and environmental factors that contribute to these differences in mortality rates.

Our study indicated excess CA‐related in patients with CKD during the first year of pandemic aligned with previously reported increased cardiovascular adverse outcomes attributable to COVID‐19.^26^, ^27^ This increased mortality could be due to the disruption of medical care with shifting resources to COVID‐19 patients, reduced in‐center dialysis utilization, and limited bystander‐initiated cardiopulmonary resuscitation due to social distancing measures.^26^, ^28^, ^29^ Additionally, the disproportionate impact of COVID‐19 on CA mortality among minority population, including Hispanic and Black patients, was likely driven by the exacerbation of existing disparities and worsening of multiple SDOH factors, such as psychosocial stress, food insecurity, and poverty.^30^

Limitations include misclassification of causes of death, ecological fallacy, and lack of individual‐level data and inability to account for different stages of CKD, which may obscure confounding factors. Although these limitations warrant cautious interpretation of our results, the use of nationally representative sample provides a reliable source for assessing trends and disparities in our study. Further research is warranted to contribute to our findings and further explore health inequities.

## 5 CONCLUSIONS

Our findings revealed that CKD‐related CA mortality rates have remained stable, with notable disparities observed among gender, racial/ethnic, and regional subpopulations. The overall trend indicated that increased SVI correlated with higher mortality rates. These results display the epidemiological inequity in CKD and cardiovascular outcomes, warranting further interventions targeting healthcare disparities.

## FUNDING INFORMATION

This research did not receive any specific grant from funding agencies in the public, commercial, or not‐for‐profit sectors.

## CONFLICT OF INTEREST STATEMENT

The authors have no conflict of interest or disclosures.

## ETHICS STATEMENT

Institutional Review Board approval was not needed as it involved deidentified and publicly available data regarding nonliving humans.

## Data Availability

Data used in this study were obtained from an already publicly available database maintained by the CDC (WONDER).

## References

[1] Shamseddin MK , Parfrey PS . Sudden cardiac death in chronic kidney disease: epidemiology and prevention. Nat Rev Nephrol. 2011;7(3):145–154. 21283136 10.1038/nrneph.2010.191

[2] Bajaj NS , Singh A , Zhou W , Gupta A , Fujikura K , Byrne C , et al. Coronary microvascular dysfunction, left ventricular Remodeling, and clinical outcomes in patients with chronic kidney impairment. Circulation. 2020;141(1):21–33. 31779467 10.1161/CIRCULATIONAHA.119.043916 PMC7117866

[3] Whitman IR , Feldman HI , Deo R . CKD and sudden cardiac death: epidemiology, mechanisms, and therapeutic approaches. J Am Soc Nephrol. 2012;23(12):1929–1939. 23100219 10.1681/ASN.2012010037 PMC3507359

[4] Ibrahim R , Sainbayar E , Pham HN , Shahid M , Saleh AA , Javed Z , et al. Social vulnerability index and cardiovascular disease care continuum: a scoping review. JACC: Adv. 2024;3:100858. 39130018 10.1016/j.jacadv.2024.100858 PMC11312302

[5] Murphy SL , Kochanek KD , Xu J , Arias E . Mortality in the United States, 2020. NCHS Data Brief. 2021;427:1–8. 34978528

[6] Steindel SJ . International classification of diseases, 10th edition, clinical modification and procedure coding system: descriptive overview of the next generation HIPAA code sets. J Am Med Inform Assoc. 2010;17(3):274–282. 20442144 10.1136/jamia.2009.001230 PMC2995704

[7] Khalid N , Afzal MA , Abdullah M , Khalil M , Shamoon YF , Abboud R , et al. Trends in cardiac arrest related mortality in the United States from 1999–2020—a CDC wonder database analysis. J Am Coll Cardiol. 2024;83(13_Supplement):2368.

[8] Collaboration, G.B.D.C.K.D . Global, regional, and national burden of chronic kidney disease, 1990–2017: a systematic analysis for the global burden of disease study 2017. Lancet. 2020;395(10225):709–733. 32061315 10.1016/S0140-6736(20)30045-3 PMC7049905

[9] Naoum P , Kitsonis D , Topkaroglou L , Skroumpelos A , Athanasakis K , Melexopoulou C , et al. ESRD patients in Greece during the financial crisis. Int J Artif Organs. 2016;39(5):258–259. 27140296 10.5301/ijao.5000495

[10] National Academies of Sciences, E., Medicine Division, Committee on Health Care Utilization, Adults with Disabilities . Health‐care utilization as a proxy in disability determination. Washington (DC): National Academies Press; 2018. 29782136

[11] Balafa O , Fernandez‐Fernandez B , Ortiz A , Dounousi E , Ekart R , Ferro CJ , et al. Sex disparities in mortality and cardiovascular outcomes in chronic kidney disease. Clin Kidney J. 2024;17(3):sfae044. 38638550 10.1093/ckj/sfae044 PMC11024840

[12] Neugarten J , Acharya A , Silbiger SR . Effect of gender on the progression of nondiabetic renal disease: a meta‐analysis. J Am Soc Nephrol. 2000;11(2):319–329. 10665939 10.1681/ASN.V112319

[13] McClellan WM , Newsome BB , McClure LA , Howard G , Volkova N , Audhya P , et al. Poverty and racial disparities in kidney disease: the REGARDS study. Am J Nephrol. 2010;32(1):38–46. 20516678 10.1159/000313883 PMC2914392

[14] Mercen JL , Curran KM , Belmar MT , Sanchez J , Hasan I , Kalra S , et al. Social determinants of health impacting access to renal dialysis for racial/ethnic minorities. Cureus. 2023;15(9):e45826. 37876398 10.7759/cureus.45826 PMC10593311

[15] Cervantes L , Robinson BM , Steiner JF , Myaskovsky L . Culturally concordant community‐health workers: building sustainable community‐based interventions that eliminate kidney health disparities. J Am Soc Nephrol. 2022;33(7):1252–1254. 35474023 10.1681/ASN.2022030319 PMC9257810

[16] Gbadegesin RA , Ulasi I , Ajayi S , Raji Y , Olanrewaju T , Osafo C , et al. APOL1 Bi‐ and Monoallelic variants and chronic kidney disease in west Africans. N Engl J Med. 2024. 10.1056/NEJMoa2404211 PMC11735277 39465900

[17] Genovese G , Friedman DJ , Ross MD , Lecordier L , Uzureau P , Freedman BI , et al. Association of trypanolytic ApoL1 variants with kidney disease in African Americans. Science. 2010;329(5993):841–845. 20647424 10.1126/science.1193032 PMC2980843

[18] Chahine M , Fontaine JM , Boutjdir M . Racial disparities in ion channelopathies and inherited cardiovascular diseases associated with sudden cardiac death. J Am Heart Assoc. 2022;11(6):e023446. 35243873 10.1161/JAHA.121.023446 PMC9075281

[19] Wang V , Wilson LE , Rowen NP , Sloan CE , Maciejewski ML , Hammill BG . Medicare Enrollment and spending among patients initiating dialysis after the affordable care act. JAMA Health Forum. 2024;5(12):e244304. 39641939 10.1001/jamahealthforum.2024.4304 PMC11624581

[20] Nieuwenhuijsen MJ . Influence of urban and transport planning and the city environment on cardiovascular disease. Nat Rev Cardiol. 2018;15(7):432–438. 29654256 10.1038/s41569-018-0003-2

[21] Barber S , Hickson DMA , Kawachi I , Subramanian SV , Earls F . Neighborhood disadvantage and cumulative biological risk among a socioeconomically diverse sample of African American adults: an examination in the Jackson Heart Study. J Racial Ethn Health Disparities. 2016;3(3):444–456. 27294737 10.1007/s40615-015-0157-0 PMC4911317

[22] Cyr ME , Etchin AG , Guthrie BJ , Benneyan JC . Access to specialty healthcare in urban versus rural US populations: a systematic literature review. BMC Health Serv Res. 2019;19(1):974. 31852493 10.1186/s12913-019-4815-5 PMC6921587

[23] Peters GA , Ordoobadi AJ , Panchal AR , Cash RE . Differences in out‐of‐hospital cardiac arrest management and outcomes across urban, suburban, and rural settings. Prehosp Emerg Care. 2023;27(2):162–169. 34913821 10.1080/10903127.2021.2018076

[24] Diez Roux AV . Residential environments and cardiovascular risk. J Urban Health. 2003;80(4):569–589. 14709706 10.1093/jurban/jtg065 PMC3456219

[25] Parcha V , Kalra R , Suri SS , Malla G , Wang TJ , Arora G , et al. Geographic variation in cardiovascular health among American adults. Mayo Clin Proc. 2021;96(7):1770–1781. 33775420 10.1016/j.mayocp.2020.12.034 PMC8260439

[26] Pham HN , Ibrahim R , Sainbayar E , Olson A , Singh A , Khanji MY , et al. Burden of hyperlipidemia, cardiovascular mortality, and COVID‐19: a retrospective‐cohort analysis of US data. J Am Heart Assoc. 2024;e037381. 39526321 10.1161/JAHA.124.037381 PMC12132682

[27] Woodruff RC , Tong X , Khan SS , Shah NS , Jackson SL , Loustalot F , et al. Trends in cardiovascular disease mortality rates and excess deaths, 2010–2022. Am J Prev Med. 2024;66(4):582–589. 37972797 10.1016/j.amepre.2023.11.009 PMC10957309

[28] Weinhandl ED . The coronavirus disease 2019 pandemic: the disruptor that maintenance dialysis never anticipated. Curr Opin Nephrol Hypertens. 2022;31(2):185–190. 35086986 10.1097/MNH.0000000000000777

[29] Stirparo G , Fagoni N , Bellini L , Oradini‐Alacreu A , Migliari M , Villa GF , et al. Cardiopulmonary resuscitation missed by bystanders: collateral damage of coronavirus disease 2019. Acta Anaesthesiol Scand. 2022;66(9):1124–1129. 35894939 10.1111/aas.14117 PMC9349817

[30] Kobo O , Abramov D , Fudim M , Sharma G , Bang V , Deshpande A , et al. Has the first year of the COVID‐19 pandemic reversed the trends in CV mortality between 1999 and 2019 in the United States? Eur Heart J Qual Care Clin Outcomes. 2023;9(4):367–376. 36442154 10.1093/ehjqcco/qcac080

